# Monitoring protein unfolding transitions by NMR-spectroscopy

**DOI:** 10.1007/s10858-021-00389-3

**Published:** 2022-01-04

**Authors:** Matthias Dreydoppel, Jochen Balbach, Ulrich Weininger

**Affiliations:** Institute of Physics, Biophysics, Martin-Luther-University Halle-Wittenberg, 06120 Halle (Saale), Germany

**Keywords:** Protein folding, Folding transition, NMR exchange regime, Global unfolding

## Abstract

NMR-spectroscopy has certain unique advantages for recording unfolding transitions of proteins compared e.g. to optical methods. It enables per-residue monitoring and separate detection of the folded and unfolded state as well as possible equilibrium intermediates. This allows a detailed view on the state and cooperativity of folding of the protein of interest and the correct interpretation of subsequent experiments. Here we summarize in detail practical and theoretical aspects of such experiments. Certain pitfalls can be avoided, and meaningful simplification can be made during the analysis. Especially a good understanding of the NMR exchange regime and relaxation properties of the system of interest is beneficial. We show by a global analysis of signals of the folded and unfolded state of GB1 how accurate values of unfolding can be extracted and what limits different NMR detection and unfolding methods. E.g. commonly used exchangeable amides can lead to a systematic under determination of the thermodynamic protein stability. We give several perspectives of how to deal with more complex proteins and how the knowledge about protein stability at residue resolution helps to understand protein properties under crowding conditions, during phase separation and under high pressure.

## Introduction

Proteins fulfil a wealth of central biological functions and the relationship between their structure and function is well established (Fersht 1977). The native and functional state, however, is usually only marginally stable towards unfolding, allowing e.g. a certain plasticity needed for function (Teilum et al. 2011) or an additional regulation via the degradation system. Protein folding can be described by energy landscapes of different complexity (Dill and Chan 1997; Oliveberg and Wolynes 2005) and often a barrier crossing leads to protein misfolding (Dobson 2003; Jahn and Radford 2005) as onset of multiple diseases.

Protein folding and its thermodynamic stability can be studied at equilibrium by changing temperature, denaturant, or pressure (Eq. 1) (Buchner and Kiefhaber 2005) and thereby changing the populations of different states (native, unfolded and intermediate) represented by the standard Gibbs free energy change upon unfolding ∆*G*°. Solvent properties such as pH or ionic strength can also shift these populations (Akasaka et al. 2013; Buchner and Kiefhaber 2005; Casares-Atienza et al. 2011; Dreydoppel et al. 2018; Scharnagl et al. 2005)

1 $${\updelta }\Delta G^\circ = { }\Delta V^\circ {\text{d}}p\,{ }{-}{ }\Delta S^\circ {\text{d}}T{ } + { }m{\text{d}}\left[ {denat} \right]$$

Such unfolding transitions are routinely monitored by fluorescence or CD spectroscopy with small amounts of protein (Buchner and Kiefhaber 2005) which can even be automated e.g. in thermal shift assays (Semisotnov et al. 1991). Alternatively, these transitions can be detected by NMR-spectroscopy which has been established over 5 decades (Ferguson and Phillips 1967; Mcdonald et al. 1971). Recently, it has become a useful approach to study protein folding under the influence of pressure (Dubois et al. 2020; Klamt et al. 2019; Roche et al. 2019; Xu et al. 2021; Zhang et al. 2019) and under crowding conditions (Köhn and Kovermann 2020), because of certain advantages of NMR-spectroscopy, that enable a more exact view on protein folding and its cooperativity also in vitro. In combination with two-dimensional detection, it allows a residue resolved view of the folding protein chain (Dyson and Wright 2004; Fossat et al. 2016). A precise knowledge about the state of folding or unfolding (including intermediates) at given conditions is a crucial requirement for more advanced investigations by NMR or other methods. For example a reduced activity of the protein can arise from the protein being not 100% native and functional any more, even if it does not show signs of the unfolded state but native like intermediates. In addition it is a requirement for understanding seemingly complex NMR spectra, that can arise from the presence of intermediate or unfolded species. Such potential folding intermediates populating at equilibrium which are otherwise hidden or remain undetected by conventional optical methods can be easily identified and structurally characterized (Balbach et al. 1996; Weininger et al. 2009). Another advantage of NMR is, that it allows the separate detection of native, unfolded and intermediate species, if the transition occurs in the slow NMR exchange regime (Löw et al. 2008). Typically all three protein states give rise to isolated NMR resonances. This separation is not observed in fluorescence or CD spectra, where the band structure of the signals of different protein conformations causes strong overlap. In addition, these bands depend on the unfolding conditions, which have to be determined additionally to the transition itself (baselines). Moreover, the cooperativity of an unfolding transition can be experimentally confirmed by analysing NMR resonances of residues from various sites. Calorimetric and optical methods can only assume cooperativity as prerequisite for the employed folding model (Buchner and Kiefhaber 2005).

Current efforts in structural biology include protein studies under conditions as close as possible to the natural environment (Selenko 2019) in cells or cell lysates (Danielsson et al. 2015; Luchinat and Banci 2018; Welte and Kovermann 2020). Here, molecular crowding becomes an issue and isotope editing of uniformly labelled protein by NMR allow thermodynamic analyses at residue resolution even in living cells and a comparison with the well studied protein under in vitro conditions can reveal local and global influences to the thermodynamic stability of the protein of interest (Danielsson et al. 2015). Intermolecular interactions driving liquid–liquid phase separation (LLPS) are especially difficult to investigate and NMR is one of few valuable methods to gain these molecular details (Emmanouilidis et al. 2021). Not only intrinsically disordered proteins but also folded and thermodynamically stable proteins drive LLPS and get analysed by NMR chemical shift analyses (Fritzsching et al. 2020). Last but not least, evolution might have started in the deep sea under high pressure conditions, which influences protein stability, LLPS and protein function (Cinar et al. 2019; Luong et al. 2015). Again, NMR reveals the molecular details of high pressure induced local unfolding of proteins (Inoue et al. 2000). In all these cases, a detailed knowledge of the protein stability at residue resolution of the system in vitro is required to compare and better understand the protein properties under in vivo conditions.

For the majority of globular small single domain proteins, the exchange between folded conformations and the unfolded state is slow on the NMR chemical shift time scale and follows a cooperative two-state folding mechanism. Deviations from these prerequisites and how to deal with them are discussed in later paragraphs. Isolated NMR resonances representing the native (*N*) or unfolded (*U*) state further simplify the analyses, because the intensity of these signals is zero under strong unfolding conditions for *N* and strong folding conditions for *U* (Dreydoppel et al. 2018; Hofmann et al. 2009; Löw et al. 2008): In this case, it is not necessary to monitor unfolding transitions until their completion for an accurate baseline determination in order to adequately extract thermodynamic parameters. NMR detected folding can thus be monitored according to

2 $$Int(N)= \frac{N0+n\cdot X}{1+{e}^{-\frac{\Delta G\mathrm{u}^\circ (X)}{RT}}}$$

3 $$Int(U)= \frac{U0+u\cdot X}{1+{e}^{+\frac{\Delta G\mathrm{u}^\circ (X)}{RT}}}$$

where *Int*(*N*) is the signal intensity of signals from the native state, *Int*(*U*) is the signal intensity of signals from the denatured state, *X* is the variable of unfolding (Eq. 1), *N*_0_ the native signal intensity at *X* = 0, *n* is the slope of the native baseline, *U*_0_ is the unfolded signal intensity at *X* = 0, *u* is the slope of the unfolded baseline and ∆*G*_u_°(*X*) is the standard Gibbs free energy of unfolding. For temperature transitions, *X* corresponds to *T* and the Gibbs free energy is defined as ∆*G*_u_°(*X*) = ∆*H°*(*T*) − *T·*∆*S°*(*T*), where ∆*H°*(*T*) and ∆*S°*(*T*) are the standard enthalpy and entropy changes upon unfolding, respectively. The temperature dependence of these parameters is determined by the heat capacity change upon unfolding, ∆*C*_p_. Upon introducing the transition midpoint *T*_m_, the temperature dependence of the Gibbs free energy of unfolding simplifies to

4 $$\Delta {G}_{\mathrm{u}}^\circ \left(T\right)=\Delta H\left({T}_{\mathrm{m}}\right)\cdot \left(\frac{{T}_{\mathrm{m}}-T}{{T}_{\mathrm{m}}}\right)+\Delta {C}_{\mathrm{p}}\cdot (\left(T-{T}_{\mathrm{m}}\right)+T\cdot \mathrm{ln}\left(\frac{{T}_{\mathrm{m}}}{T}\right))$$

For denaturant induced unfolding ∆*G*_u_°(*X*) = ∆*G*_u,H2O_°−*m*·[*denat*] with ∆*G*_u,H2O_° representing the standard Gibbs free energy of unfolding at denaturant concentration [*denat*] of 0, and *m* describes the cooperativity of the transition. The analysis according to Eqs. 2 and 3 implies a two-state protein folding mechanism $N\leftrightarrow U$ and the midpoint of transition is reached when ∆*G*_u_°(*X*) = 0.

In order to extract accurate thermodynamic parameters from equilibrium folding transitions the correct analysis has to be performed, which is dependent on the actual NMR detection method, the method of unfolding and the exchange regime. Additionally the correct model has to be chosen. While this is possible for kinetic protein folding experiments (Löw et al. 2007), equilibrium unfolding is usually limited to the simple and robust two-state model. Since in NMR different states give rise to different signals in the NMR spectra, transitions can be observed not only between *N* and *U*, but also to intermediate states. As a consequence even more complex folding behaviours can be captured by extended simple models (Löw et al. 2008).

Here, we summarize NMR specific aspects for the analysis of folding transitions in terms of different methods of unfolding, different NMR time regimes and different NMR probes. We conduct differently induced (urea, GdmCl, temperature) unfolding transitions of the model protein GB1 detected in 2D by amide protons and methyl groups, as well as in proton 1D experiments in order to illustrate these different aspects of protein unfolding transitions followed by NMR-spectroscopy. Advantages and limitations of the subsequent analyses towards thermodynamic parameters are discussed for the different NMR methods. We give perspectives of how to deal with more complex conditions and systems compared to in vitro GB1 unfolding.

### Practical aspects of acquiring NMR unfolding transitions

Proteins can typically be unfolded by changing temperature, including cold denaturation (Adrover et al. 2010), pressure (Roche et al. 2017), or increasing the concentration of denaturant (usually urea or GdmCl and for thermodynamically very stable proteins GdmSCN (Zeeb et al. 2004), (see Eq. 1). In rare cases also changes in pH (Balbach et al. 1995; Dyson and Wright 2017) or salt (Mücke and Schmid 1994) can be used. Unfolding induced by temperature and pressure have the principle advantage that just one sample is required and the protein concentration is identical in all measurements if the system does not suffer from aggregation. For denaturant (or pH) induced unfolding several different samples are needed. An identical protein concentration in all samples is best achieved by preparing two samples from the same protein stock at the extreme conditions (low and high pH, salt or denaturant) and creating all other samples by mixing the initial ones (Greene et al. 2006; Hofmann et al. 2009). Exact pH or denaturant concentration has to be determined after each point (usually by taking small aliquots from these samples). In all cases it is necessary to wait till equilibrium has been reached, which usually is after a few seconds but can take years in rare cases (Puorger et al. 2008). For quantitative analyses based on the two-state model, full reversibility of the folding and unfolding reaction is required. Therefore it is beneficial to acquire the folding transition from at least two separate samples, one under folding and the other under unfolding conditions. By changing the conditions so that both samples approach the conditions of the midpoint of unfolding, half a folding and half an unfolding transition can be acquired. Both transitions contain NMR resonances of the native and unfolded state. So, reversibility can be tested, if the extrapolated entire transition curves of the native and unfolded state from both halves overlap (Hofmann et al. 2009).

**Fig. 1:**
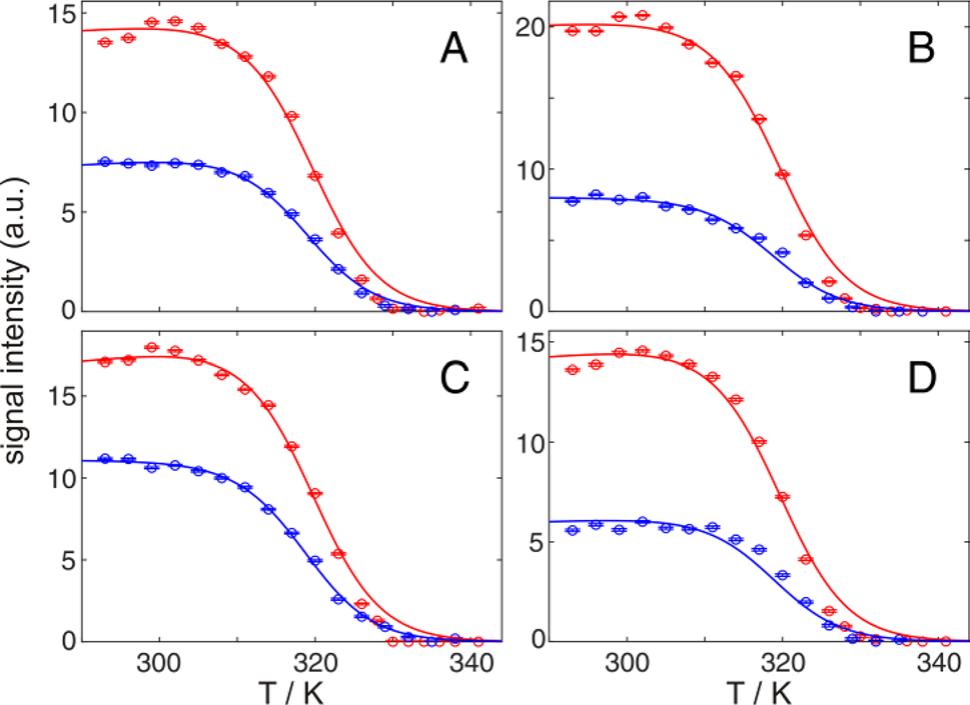
Temperature induced unfolding (red) and refolding (blue) of 1 mM GB1 in 20 mM HEPES, pH 7.1. Representative raw intensity data of the transitions monitored by the native state methyl group NMR signals of L5δ1 (**A**), L7δ2 (**B**), V29γ1 (**C**), and V54γ1 (**D**) recorded in 2D ^1^H^13^C HMQC spectra. Solid lines represent a global fit according to Eqs. 2 and 3 (two-state model) applied to all observed methyl groups of the native and unfolded state. Midpoints of the unfolding and refolding transitions are 319.3 ± 0.1 K and 318.6 ± 0.1 K, respectively. By refolding only 40–70% of the initial intensity can be achieved

Temperature induced transitions are most problematic in terms of aggregation (especially at high NMR concentrations) and reversibility has to be checked (e.g. by acquiring unfolding and refolding transitions). A sigmoidal looking unfolding transition is not enough to verify reversibility. As demonstrated by temperature induced un- and refolding of GB1 (Fig. 1) the intensity difference after refolding is high, pointing to high losses because of aggregation at high temperatures. Nevertheless, while the whole system is clearly not reversible, transition midpoints between unfolding and refolding are pretty similar. This can be explained by a refolding transition with simply a lower protein concentration. In other words the aggregated protein does not contribute much to the monitored transition curves, since it is simply not visible by NMR. Thereby the problem of reversibility, while still there, is often reduced.

Pressure induced unfolding requires a high pressure NMR device (Klamt et al. 2019) and the feasible pressure range up to 300 MPa causes often only slight shifts of the folding equilibrium. Unfolding induced by pH variation can lead to aggregation at certain pH values (e.g. near the isoelectric point) and to a drastic increase of ionic strength (below pH 2 and above pH 12) that causes a loss of spectrometer performance and thus artificially reduced signal intensities (Raum and Weininger 2019). Additionally there is no single buffer system to cover the whole pH range, therefore one has to use different buffer systems or (if possible) a self buffering protein in water (Wallerstein et al. 2015). High levels of ionic strength are also introduced by GdmCl, thus urea is the better suited denaturant for NMR, although it is the weaker denaturant. A high ionic strength and therefore increased conductivity of the sample reduces signal-to-noise of the detection coil. Especially cryogenically cooled probes suffer from this losses. While this can be partially compensated by using 3 mm NMR tubes (Voehler et al. 2006) and salt tolerant cryo probes (Robosky et al. 2007) it is insufficient for the here used GdmCl concentrations of up to 5 M. Therefore a room temperature probe is recommended. Both for urea and GdmCl induced denaturation, signals of the denaturant have to be suppressed, ideally in the case of ^15^ N and/or ^13^C labelled samples by gradient selection (Löw et al. 2008), in the case of unlabeled samples by additional pre-saturation, selective excitation or simply measuring in D_2_O and deuterated denaturant. Alternative methods include ^19^F substituted residues and protein ^19^F NMR-spectroscopy (Frieden 2007).

### NMR exchange regimes

**Fig. 2:**
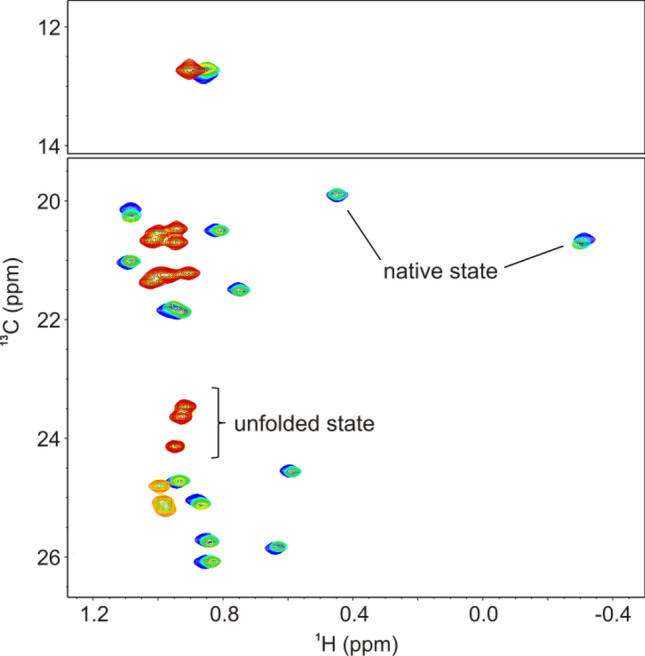
Methyl region of 2D ^1^H^13^C HMQC spectra of 900 µM GB1 in 20 mM HEPES, pH 7.0 at 300 K and urea concentrations of 0 M (blue), 2.9 M (cyan), 4.05 M (green), 5.4 M (yellow), and 6.52 M (red). Representative cross peaks of the native state and unfolded state are indicated

NMR signals from different species at equilibrium (such as the folded and unfolded state) can occur in three different exchange regimes (Palmer et al. 2001) and thus are affected differently in unfolding transitions. Note that these regimes can change during the transition, e.g. at different temperatures. In the slow exchange regime the exchange rate between two different states is much smaller than the chemical shift difference (in Hertz) of the corresponding signals in exchange. Both states will give rise to independent signals (Fig. 2) and their intensity is directly linked to the population and relaxation properties of each moiety. Most proteins unfold in this regime, since chemical shift differences between native and unfolded states are usually large and proteins fold/unfold relatively slowly. Even at refolding rates of 1000 s^−1^ a slow exchange regime has been observed (Schindler et al. 1995; Zeeb and Balbach 2005b), and typically folding rates decrease towards the unfolding transition midpoints.

In contrast, the fast exchange regime corresponds to exchange rates between two different states much faster than the chemical shift difference (in Hertz) of the corresponding signals. Thus one will only detect average signals between the two different states and the chemical shift position of this average signal is directly linearly linked to the population. Only the fastest folding processes fall in this category (Sadqi et al. 2006; Wang et al. 2003).

If the exchange rate between two different states is similar to the chemical shift difference of the corresponding signals in exchange the intermediate exchange regime applies. Neither chemical shifts nor intensities are linearly linked to the populations any more. Under these circumstances it is recommended to use more advanced kinetic NMR experiments and analyses to derive folding rates e.g. by line shapes (Wang et al. 2003; Zeeb and Balbach 2005a) or relaxation dispersion methods (Korzhnev and Kay 2008; Palmer 2004; Weininger et al. 2012; Zeeb and Balbach 2005b). Since the exchange regime depends on the chemical shift difference, different signals can be in different exchange regimes although the folding rates are the same. Therefore it is advisable to always critically check the exchange regime in which the observed transitions occur, as discussed below.

### NMR detected unfolding transitions in the slow exchange limit

**Fig. 3:**
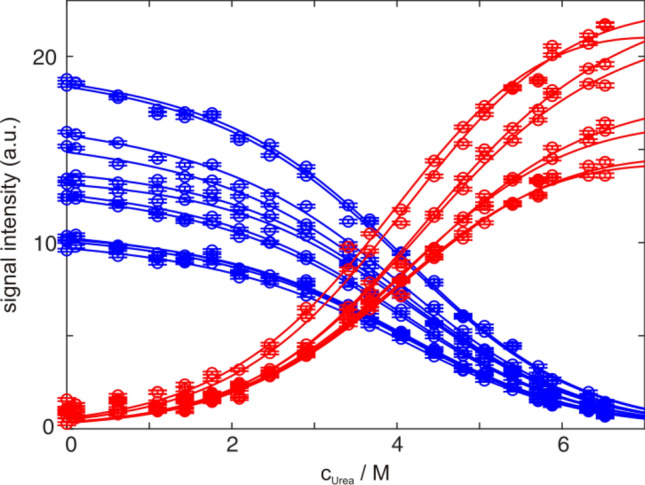
Urea induced unfolding of 900 µM GB1 in 20 mM HEPES, pH 7.0 at 300 K. Raw intensity data of methyl groups in 2D ^1^H^13^C HMQC spectra of the native state (blue dots) and the unfolded state (red dots), together with the global fit (solid line according to Eqs. 2 and 3) are plotted against the urea concentration

The slow exchange limit is by far the most observed case for protein folding. There are different signals from the native, unfolded and possibly intermediate species in the spectra. Going from native to unfolding conditions signals of the sufficiently stable native state will decrease to zero, signals from possible intermediate states will rise from zero and decrease back to zero, while signals from the unfolded state will increase from zero (Löw et al. 2008). Changes in the signal intensities (or volumes) directly reflect changes in populations. However populations cannot simply be estimated by building ratios of signal intensities from different states (with the exception of one pulse 1D experiments with good S/N and fully relaxed longitudinal magnetization before excitation), since their relaxation properties are very different. The signal intensity for native state signals can be significantly reduced compared to signals from the unfolded state because of relaxation losses during the pulse sequence (Fig. 3), even for methyl groups of small proteins like GB1. In other words signals from the native state (or even within) have a different strength than signals from the unfolded state. Note that changes of these signals during the transition still are direct reflections of changes in populations (e.g. visualized after normalization) (Szyperski et al. 2006). When applying a two-state model for fitting (Eqs. 2 and 3), linear baselines imply that all factors determining the NMR signal of one state depend linearly on the denaturant or temperature, which is only an assumption (see below).

### Restrictions of baselines

**Fig. 4:**
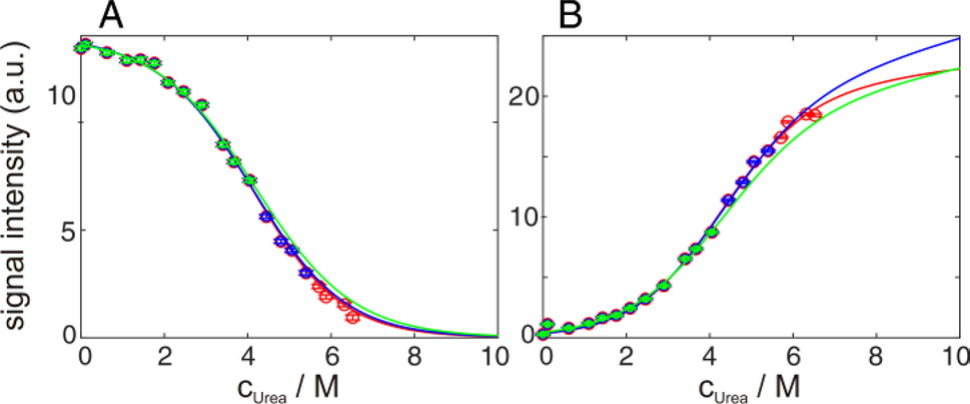
Urea induced unfolding of 900 µM GB1 in 20 mM HEPES, pH 7.0 at 300 K. Representative raw intensity data from 2D ^1^H^13^C HMQC spectra of V21γ2 (**A**) and an unassigned methyl group of the unfolded state (**B**), fitted globally with all other methyl signals according to Eqs. 2 and 3, using all 20 measured data points (red), only the first 16 (blue) or only the first 12 (green) data points of all signals. Results of the fits are summarized in Table 1

During the whole transition, the unfolded baseline of well isolated native state signals is zero, as is the native baseline of well isolated unfolded state signals. For proteins with a thermodynamic stability of ∆*G*_u_° > 10 kJ/mol the native baseline is reached and can be experimentally observed in the absence of denaturant, because the corresponding equilibrium constant *K* = exp(−∆*G*_u_°/*RT*) drops below 0.02. Both baselines for possible intermediate signals are zero as well (Löw et al. 2008). Thus the number of parameters that need to be determined from an unfolding transition is reduced compared to optical detection methods. In the latter case, these baselines need to be determined from the signal change at the beginning and end of the unfolding transition, where population changes are neglectable (Buchner and Kiefhaber 2005). Furthermore, one does not necessarily have to achieve 100% unfolding for fitting native state signals and one does not have to start at 100% folded when fitting unfolded state signals, as can been seen in Fig. 4 and Table 1 with realistic deviations for the derived thermodynamic parameter from sparse data points. Additionally, the slope of the baseline of the native state (*n* in Eq. 2) and of the baseline of the unfolded state (*u* in Eq. 3) can be restricted (positive or negative slope) in some cases, since it is dependent on changes of protein dynamics.

**Table 1:** Global fit of urea induced unfolding of GB1, detected by selectively ^13^C labeled methyl groups for transitions of a different completeness as depicted in Fig. 4

Data set	State	Midpoint (M urea)	*m*-Value (kJ/mol/M)	∆*G* (kJ/mol)
20 Points	*N*	4.43 ± 0.05	2.56 ± 0.02	11.3 ± 0.1
	*U*	3.91 ± 0.03	2.37 ± 0.01	9.3 ± 0.1
	*N* + *U*	4.20 ± 0.02	2.37 ± 0.01	9.9 ± 0.1
16 Points	*N*	4.36 ± 0.08	2.45 ± 0.03	10.7 ± 0.2
	*U*	3.70 ± 0.08	2.11 ± 0.02	7.8 ± 0.2
	*N* + *U*	4.15 ± 0.05	2.26 ± 0.01	9.4 ± 0.1
12 Points	*N*	4.35 ± 0.21	2.39 ± 0.07	10.4 ± 0.4
	*U*	3.83 ± 0.14	2.08 ± 0.05	8.0 ± 0.2
	*N* + *U*	4.17 ± 0.12	2.12 ± 0.03	8.8 ± 0.2
Fluorescence	Averaged	4.06 ± 0.11	2.68 ± 0.05	10.9 ± 0.2

Errors are estimated by Monte-Carlo simulations and do not include systematic errors

**Fig. 5:**
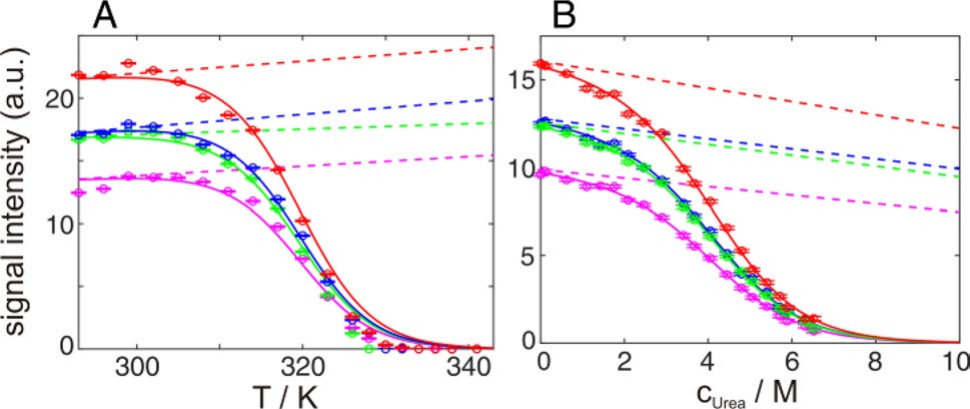
Baselines (dashed lines) of temperature (**A**) and urea (**B**) induced unfolding transitions of GB1, monitored on several methyl groups in 2D ^1^H^13^C HMQC spectra of the native state (L7δ1 red, V29γ1 blue, V39γ1 green, V54γ2 magenta). Raw intensities are shown as dots, the global fit according to Eq. 2 as a solid line

In the case of unfolding induced by temperature increase the baselines are constant or rising depending on the influence of the decreasing protein rotational correlation time with temperature (slopes *n* and *u* ≥ 0 in Eqs. 2 and 3), due to decreased viscosity of the solvent and higher molecular mobility (Fig. 5A). For pressure things are more complex since viscosity decreases with pressure below 33 °C and increases above. Thus baselines are expected to slightly increase (below 33 °C) or decrease (above 33 °C) with pressure. However the viscosity effects of pressure are much smaller compared to the viscosity effects of temperature and baselines are typically considered to be constants (*n* and *u* ~ 0 in Eqs. 2 and 3) with increasing pressure (Fossat et al. 2016). In the case of chemical denaturants such as urea or GdmCl the viscosity increases with their concentration resulting in sloping or constant (*n* and *u* ≤ 0 in Eqs. 2 and 3) baselines (Haupt et al. 2011) (Fig. 5B), because the reduced rotational tumbling promotes relaxation losses during 2D NMR correlation experiments. Quantifications of the latter effects are complex and therefore non linear baselines are typically not applied. Often only changes of the transition midpoints are discussed, where baseline effects cause only marginal shifts.

### Validation of the slow exchange regime

**Fig. 6:**
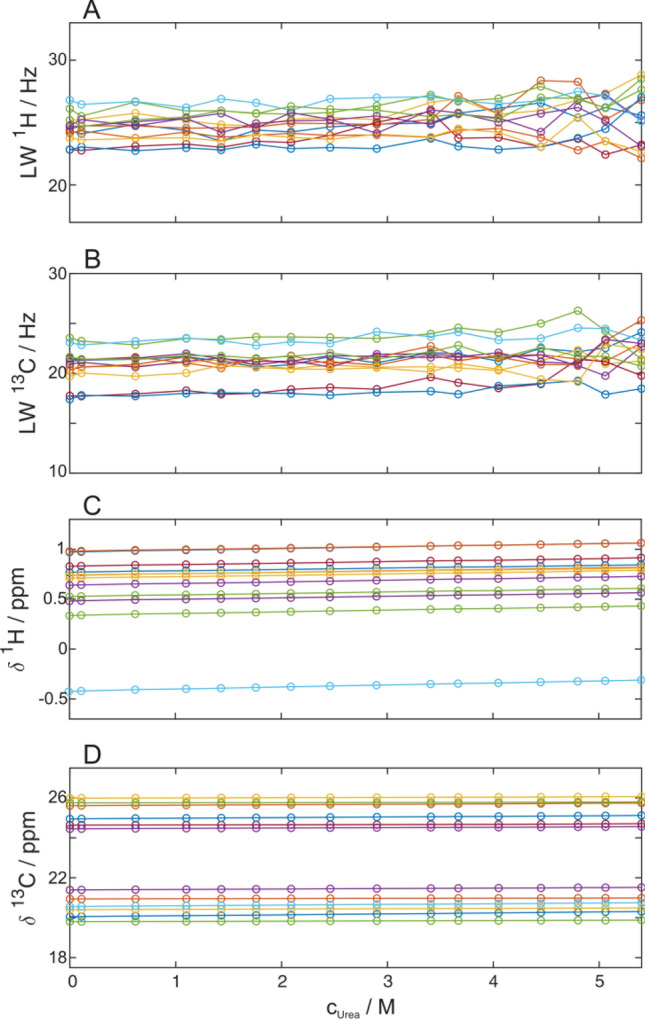
Urea induced unfolding of 900 µM GB1 in 20 mM HEPES, pH 7.0 at 300 K. ^1^H (**A**) and ^13^C (**B**) line widths and ^1^H (**C**) and ^13^C (**D**) chemical shifts of several native state methyl groups in 2D ^1^H^13^C HMQC spectra are plotted against the urea concentration

In order to verify the slow exchange limit one has to critically check linewidths and chemical shift changes for all individual signals under study. Line widths should stay the same or change uniformly according to changes in viscosity/mobility (decreased/increased line width for decreased/increased viscosity) during the unfolding transition (Fig. 6). Also ideally the chemical shifts should not change and only intensities should vary. Since the chemical shifts are often affected by changing conditions such as increasing denaturant concentrations, they should be affected uniformly in size and direction which would simply mean incorrect re-referencing of the NMR spectra by DSS. Moreover, there may be local buffer effects that could influence the chemical shifts of individual residues (most common for amide signals). These residues should be excluded at first from the global analysis of the cooperative two-state unfolding analysis (see below how excluded residues sill can contain valuable information). Knowing the regions of unfolded signals for different positions one can check for slow exchange even more precisely (Fig. 2). If all native signals are shifting towards the expected random coil chemical shifts of unfolded signals during the unfolding transition, the assumption of the slow exchange regime is not valid (Klamt et al. 2019). Another useful control is to acquire spectra under the same conditions at two magnetic field strengths. In the slow exchange regime signals are expected to display exactly the same chemical shifts. If signals appear at slightly different positions at the different field strengths it points to intermediate exchange (Skrynnikov et al. 2002).

### 1D versus 2D detection

**Fig. 7:**
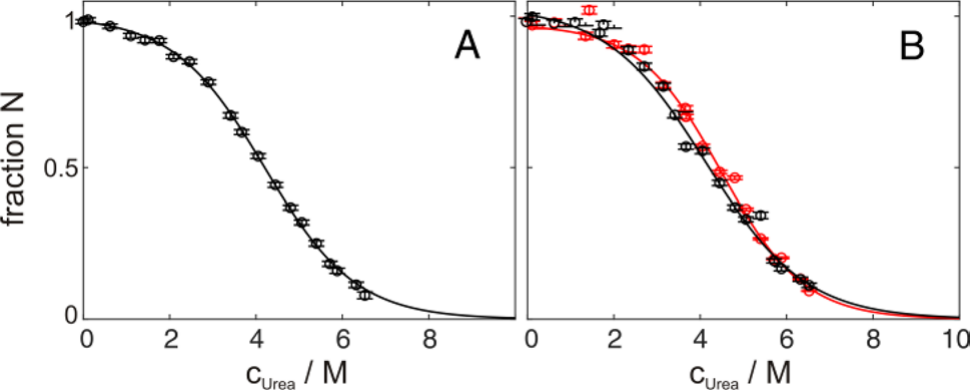
Urea induced unfolding of 900 µM GB1 in 20 mM HEPES, pH 7.0 at 300 K. (**A**) Global fit (solid line) of all native and unfolded methyl group signals, derived from ^1^H^13^C HMQC spectra, together with the baseline corrected data points of V39γ1. Fit using Eq. 2 results in a transition midpoint of (4.20 ± 0.02) M urea, an *m*-value of (2.37 ± 0.01) kJ/mol/M and a Gibbs energy of unfolding of (9.9 ± 0.1) kJ/mol. (**B**) Fraction of native protein derived from 1D proton spectra, using ^13^C-decoupling and water suppression via WATERGATE (black) or selective excitation (red). Fit results here are transition midpoints of (4.10 ± 0.01) and (4.50 ± 0.01) M urea, *m*-values of (2.20 ± 0.03) and (2.60 ± 0.2) kJ/mol/M and Gibbs energies of unfolding of (9.0 ± 0.2) and (10.2 ± 0.1) kJ/mol, respectively

Protein unfolding transitions monitored by NMR-spectroscopy are usually detected by 1D proton or 2D ^1^H^15^N or ^1^H^13^C HSQC spectra (or variants thereof). Usually 2D detection is advantageous. It allows well resolved per-residue detection and provides resolved signals of all individual (native, unfolded, intermediate) states. Thus it allows an easy identification and characterization of equilibrium intermediates (Löw et al. 2008). Furthermore, the validation of the exchange regime is also more straightforward because most of the NMR resonances are well resolved and not overlapping. The drawback of 2D detection is reduced sensitivity. Thus in certain cases it can be beneficial to use 1D detection which provides higher sensitivity. Protein folding studied by 1D NMR also enables the distinct observation of the folded and unfolded state in slow exchange in certain approaches, i.e. using His ^1^Hε1 signals in D_2_O (Dobson and Evans 1984) or ^19^F labeled aromatic amino acids (Ropson and Frieden 1992). If the system is a well established two-state folder in the slow exchange regime an all-atom view is not needed to determine global thermodynamic parameters of the investigated protein (Szyperski et al. 2006). Only signals from the native state can be monitored separately by 1D ^1^H detection in H_2_O samples, whereas resonances of the unfolded state overlap with native signals. This problem can be mathematically overcome by normalization of integrated spectral section containing only native signals and sections containing both native and unfolded signals, if the denaturation variable *X* (Eqs. 2 and 3) is known or iteratively determined for maximal protein stability (Szyperski et al. 2006). This approach has been applied to GB1 and the results are depicted in Fig. 7B. In general 1D detection is able to extract the correct thermodynamic parameters with minor problems. When using water suppression by WATERGATE, this approach slightly overestimates the population of the unfolded state, because of its favorable relaxation properties that lead to more intense signals. Therefore, a slightly lower stability and transition midpoint are detected compared to optical methods. Applying 1D detection with selective excitation of methyl groups does not run into this problem, but more practical problems with baseline correction and phasing might occur, resulting in higher data scatter but still meaningful transitions (Fig. 7). A comparison of the thermodynamic parameters derived from 1D NMR unfolding approaches with optical techniques allows to verify whether the protein behaves the same at the much higher NMR concentrations.

### Exchangeable protons

**Fig. 8:**
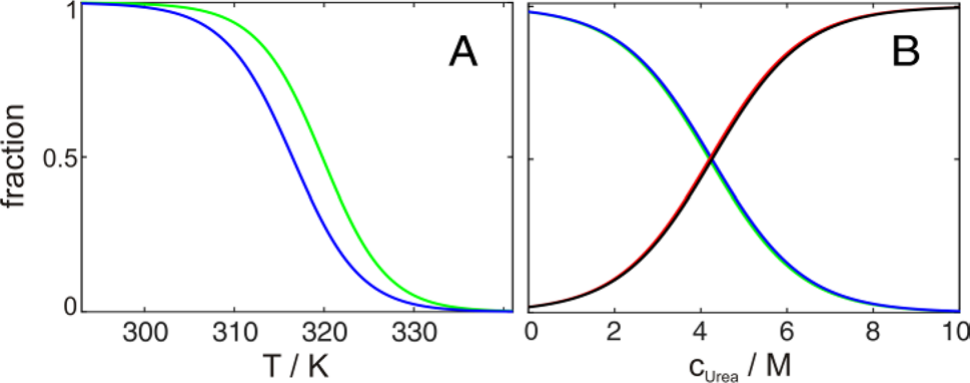
Comparison of global fits of temperature (**A**) and urea (**B**) induced unfolding of GB1 derived from methyl and amide groups. Native fractions of methyl groups are shown in green, of amide groups are shown in blue. Unfolded fractions of methyl groups are shown in red and of amide groups in black. Differences in transition midpoints are **A** 3.2 K and **B** < 0.1 M

Analyses and interpretation of NMR detected folding transitions can get more complicated for the exchangeable amide protons if amide exchange rates get sizable (at high pH or temperature). Then NMR signal intensities can decay by reduced protection from exchange with the solvent and not just by a change in population (Löw et al. 2009). This will result in sloping native baselines of the native state, because the exchange rate will increase with proceeding unfolding making restrictions of the baseline slopes more difficult. Furthermore, in extreme cases it can lead to transitions artificially shifted towards less denaturizing conditions (Fig. 8A). Additionally signals of the unfolded state (no protection from amide exchange) will be severely reduced in intensity and might not be detectable at all (Löw et al. 2009). These effects can artificially cause transitions of a two-state folder, detected by signals of the native and unfolded state, to not cross at 50% population (Fig. 8A). While detection by ^1^H^15^N HSQC is very common and provides several advantages such as cheap and robust labeling, straightforward resonance assignment and high spectral dispersion, these possible complications have to be considered.

### Urea versus GdmCl induced transitions

**Fig. 9:**
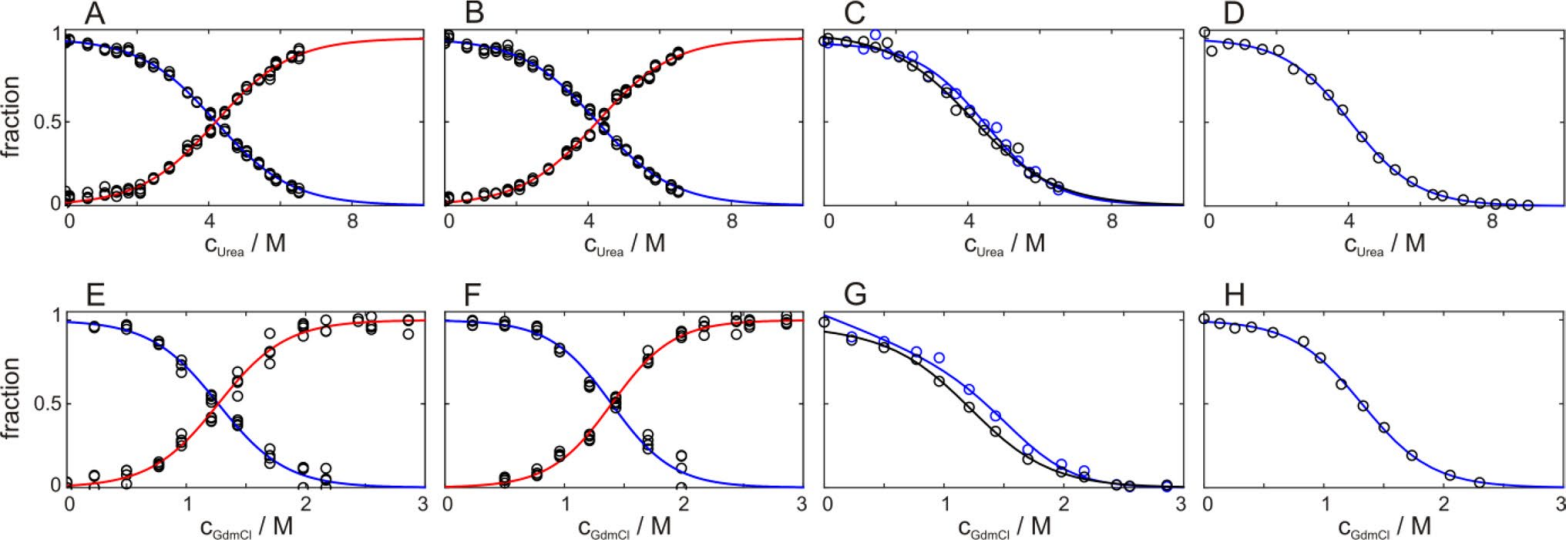
Comparison of all performed fits of GB1 unfolding, induced by urea (**A**–**D**) and GdmCl (**E**–**H**). Underlying data from selectively ^13^C labeled methyl groups (**A**, **E**), amide groups (**B**, **F**), 1D proton spectra (C,G; WG black, SE blue) and fluorescence measurements (**D**, **H**), respectively. Data points of four signals of the native state (**A**, **E** L7δ1, V29γ1, V39γ1 and V54γ2; B,F: I6, E27, D37 and E56) and of the unfolded state are shown as examples. Red lines correspond to signals from the unfolded state, which were fitted globally together with the respective native signals (blue lines). Parameters derived from fitting to Eqs. 2 and 3 are summarized in Table 2

Both urea and GdmCl induced unfolding transitions give similar results within the margin of error (Fig. 9 and Table 2). GdmCl as denaturant results in slightly higher thermodynamic stability, which can be attributed to the stabilizing effect of higher ionic strength. All urea transitions (except 1D selective excitation) reveal transition midpoints of 4.1–4.2 M urea, and thermodynamic stabilities of 10–11 kJ/mol. The 1D approach with WATERGATE results in a slightly reduced thermodynamic stability in agreement with considerations mentioned before. In the case of GdmCl all transition midpoints (except 1D selective excitation) are around 1.3 M GdmCl and derived thermodynamic stability is around 11–14 kJ/mol. Again the 1D approach with WATERGATE results in a reduced stability. Differences between the detection methods have been discussed in more detail above.

**Table 2:** (Global) fit of urea and GdmCl induced unfolding of GB1, detected by selectively ^13^C labeled methyl groups, amide groups, methyl groups in 1D by water gate (WG) and selective excitation (SE), and fluorescence

Denaturant	Detection method	Midpoint (M)	*m*-Value (kJ/mol/M)	∆*G* (kJ/mol) @ 300 K
Urea	Methyl 2D	4.20 ± 0.02	2.37 ± 0.02	9.9 ± 0.1
	Amide 2D	4.26 ± 0.02	2.35 ± 0.01	10.0 ± 0.1
	Methyl 1D WG	4.1 ± 0.1	2.17 ± 0.04	9.0 ± 0.2
	Methyl 1D SE	4.5 ± 0.1	2.61 ± 0.02	11.7 ± 0.1
	Fluorescence	4.1 ± 0.1	2.68 ± 0.05	10.9 ± 0.2
GdmCl	Methyl 2D	1.27 ± 0.01	8.82 ± 0.04	11.2 ± 0.1
	Amide 2D	1.39 ± 0.01	9.65 ± 0.04	13.5 ± 0.1
	Methyl 1D WG	1.24 ± 0.03	8.9 ± 0.1	9.3 ± 0.2
	Methyl 1D SE	1.66 ± 0.03	7.5 ± 0.1	14.7 ± 0.2
	Fluorescence	1.32 ± 0.02	8.60 ± 0.10	11.3 ± 0.2

Errors are estimated by Monte-Carlo simulations and do not include systematic errors

It should be noted that when using GdmCl, NMR intensities have to be corrected for reduced detector performance at high ionic strength. Both NMR excitation and detection are affected by the amount of ionic strength in the sample. In the case of excitation this can be compensated by a longer pulse (imperfections of longer pulses are neglected). Intensity losses from reduced detection can be corrected by a first approximation, that the 90° ^1^H pulse length is proportional to such loses (Godecke et al. 2013; Holzgrabe 2010).

$$Int_{real} = Int_{measured} \;90^{ \circ } (0^{ \circ } {\text{M}})/90^{ \circ } (x{\text{M}})$$

However, this often is not needed in practice. If fitted globally for native and unfolded state, methyl groups derived ∆*G* values with (11.2 ± 0.1 kJ/mol) and without correction (10.6 ± 0.1 kJ/mol) are very similar. The problem then is compensated by artificially steepened baselines.

In the case of the GdmCl transition the native baseline is less defined compared to the urea transition, since GB1 is a protein of low stability. For proteins with high stability the unfolded baseline would be less defined (most often seen for urea induced unfolding, for proteins with even higher stability also for GdmCl induced unfolding). However, this does not impact results, if both native and unfolded states are analyzed in a global fashion. For very stable proteins, GdmSCN is an alternative denaturant (Zeeb et al. 2004).

### Temperature transition

**Fig. 10:**
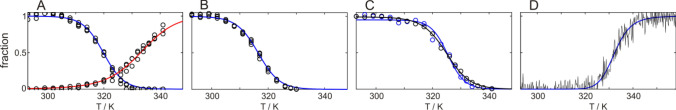
Comparison of all performed fits of GB1 temperature unfolding. Underlying data from native state (blue) and unfolded state (red) selectively ^13^C labeled methyl groups (**A**), native state amide groups (**B**), 1D proton spectra (**C**; WATERGATE black, selective excitation blue) and circular dichroism measurements (**D**), respectively. Data points of four signals of the native state (**A**: L7δ1, V29γ1, V39γ1 and V54γ2; **B**: A20, K28, F52 and E56) and of the unfolded state are shown as examples. Parameters derived from fittings to Eqs. 2 and 3 are summarized in Table 3

Temperature induced unfolding has many potential issues, even if the transition curves look reasonable (Fig. 10). One main issue is aggregation at high temperature and therefore not achieving reversibility (Fig. 1) especially under the high concentrations typically required for NMR compared to optical methods. This is less reflected in the transition midpoint, but more in the enthalpy change (Table 3). Together with extrapolation problems (the enthalpy of unfolding is determined for the midpoint of unfolding, and expected to change with temperature), this leads to often less accurate quantitative results. Moreover, a correct determination of the heat capacity change upon unfolding, ∆*C*_p_, is required for proper calculation of the transition midpoint and enthalpy change according to Eq. 4. Since ∆*C*_p_ is not sufficiently described by NMR detected temperature transitions with few exceptions (Szyperski et al. 2006), it should be provided from independent calorimetric measurements. For GB1, we used a value of 2.34 kJ/mol/K from DSC experiments (Dreydoppel et al. 2018), which was set fixed for the analysis.

**Table 3:** (Global) fit of temperature induced unfolding of GB1, detected by selectively ^13^C labeled methyl groups (from native and unfolded state), amide groups (native), methyl groups in 1D by WATERGATE (WG) and selective excitation (SE) and CD spectroscopy

Direction	Detection method	Midpoint (K)	∆*H* (kJ/mol)	∆*G* (kJ/mol) @ 300 K
Heat	Methyl 2D *N*	319.3 ± 0.1	235.6 ± 0.6	13.1 ± 0.5
	Methyl 2D *U*	333.1 ± 0.1	148.0 ± 1.4	10.7 ± 1.5
	Amide 2D	316.4 ± 0.1	219.9 ± 0.4	10.4 ± 0.2
	Methyl 1D WG	324.9 ± 0.1	208.8 ± 1.0	13.7 ± 1.0
	Methyl 1D SE	325.4 ± 0.1	296.4 ± 0.8	20.7 ± 1.3
	CD	331.9 ± 0.1	285.0 ± 0.7	23.7 ± 1.7
Cool	Methyl 2D *N*	318.6 ± 0.1	197.0 ± 0.7	10.2 ± 0.4
	Methyl 2D *U*	333.5 ± 0.2	149.0 ± 2.0	10.9 ± 2.2
	Amide 2D	315.9 ± 0.1	202.0 ± 0.7	9.3 ± 0.3
	Methyl 1D WG	320.5 ± 0.1	160.8 ± 3.4	7.2 ± 2.3
	Methyl 1D SE	325.0 ± 0.1	223.3 ± 5.2	10.5 ± 0.1
	CD	329.5 ± 0.1	178.5 ± 0.5	12.8 ± 0.5

∆*C*_*p*_ is fixed to 2.34 kJ/mol/K. Errors are estimated by Monte-Carlo simulations and do not include systematic errors

Furthermore, often intermediate folding states are populated at higher temperature (Casares-Atienza et al. 2011). Therefore midpoints derived from signals of the native and the unfolded state are not equal (Fig. 10A). In GB1 we obtain three different groups of results. Midpoints from native state methyl signals are around 320 K (midpoints from amides are lower, see Fig. 8), midpoints from methyl groups from 1D experiments (based on *N* and *U* signals) are around 325 K, while midpoints from unfolded state methyl signal, together with the CD experiment, are around 330 K. The result from the 1D experiments directly arises from the combined analysis of *N* and *U* signals. One might conclude that the result based on signals from the unfolded state is the correct one, since it coincides with the CD experiment. However derived ∆*G* values (by *U* signals and CD) are far too high, compared to urea and GdmCl induced unfolding (Table 2). Here the results based on native state signals give the best agreement. Furthermore, they directly monitor the disappearing of the native state, which is the relevant state for protein function, while *U* signals and CD likely report the loss of residual structure, e.g. the helix. Taken together temperature induced unfolding is problematic, especially at high NMR concentrations. However NMR-spectroscopy is perfectly suited to spot inconsistencies and misleading interpretations.

### NMR detected unfolding transitions in the fast exchange limit

Only very fast folding proteins display transitions in the fast exchange regime (Farber et al. 2010; Sadqi et al. 2006). Here, many aspects are different and some advantages of using NMR-spectroscopy to monitor folding transitions are lost. Only averaged signals of the native and unfolded (and possibly intermediate) states are observed and the information of populations is connected to the chemical shift, which can be determined far more accurately than intensities. In this exchange regime a complete determination of the transition, including both baselines, is needed in order to unravel all the thermodynamic parameters. This is in close analogy to e.g. fluorescence detected folding transitions. An accurate determination of an unfolding transition is hard to achieve for very fast folding proteins (rates above 100,000 s^−1^) since they usually experience a low enthalpy of unfolding and therefore undergo very uncooperative unfolding. Furthermore, it is challenging to validate the fast exchange regime. The critical requirement is the absence of line broadening in the middle of the transition. However slight line broadening will not have huge effects on the transition curve. The remaining advantage of NMR-spectroscopy is an all-atom view which allows identification of equilibrium intermediates and check for barrier limited or barrier less folding. Therefore it is highly recommended to combine these transitions with 2D detection. Since all information is connected to the chemical shift, amide exchange is not a fundamental problem. Finally different sub states (potential folding intermediates) can be explored by transitions in the fast exchange regime usually in combination with pressure (Kalbitzer 2015).

### NMR detected unfolding transitions in the intermediate exchange regime

If protein folding occurs at the intermediate exchange regime only qualitative interpretations are feasible, since neither chemical shifts nor intensities are directly correlated to populations, and therefore the analyses for the slow and fast exchange limit cannot be directly transferred. In general it might be better to gain information of the populations and kinetics by dynamic line shape analyses (Wang et al. 2003) or relaxation dispersion methods (Palmer 2004; Weininger et al. 2012; Zeeb and Balbach 2005b).

### Cooperativity of protein unfolding

During the analysis of data from calorimetry and optical methods a protein folding model has to be defined to derive thermodynamic parameters from curve fittings. These models typically assume cooperative folding transitions (Buchner and Kiefhaber 2005). NMR spectroscopy has the advantage that this assumed cooperativity can be verified at residue resolution. A global analysis of unfolding transitions of all accessible residues allow to identify core residues cooperatively following one global unfolding transition. For these residues, the midpoint of unfolding transitions from NMR resonances of the native state corresponds to the midpoint of refolding from resonances of the unfolded state. In the case of the here studied GB1, all detected residues fulfil this requirement and therefore GB1 can be treated as one cooperative folding unit. Larger proteins e.g. with extended loops or two and more domains might deviate from this scenario. Local interactions with the denaturant and thus induced non-cooperative conformational changes might occur and can be easily identified by deviating from the global unfolding transition. Several examples are discussed in a recent review (Politou et al. 2021). In these cases the concept of cooperative folding still holds, since a cooperative folding unit exists and on top local effects can be identified and interpreted. In the case of the two-domain protein SlyD, non-cooperative residues from the less stable domain became obvious from very steep native baselines in comparison to the unfolding transitions of the more stable domain (Haupt et al. 2011). After these steep baselines the unfolding transitions followed global unfolding indicating that both domains form a cooperative folding unit.

## Conclusions

With the here presented data of GB1 following a two-state folding model we show how incorporation of knowledge about NMR relaxation and exchange allows for a robust analysis of NMR detected protein unfolding transitions, as well as avoiding certain misinterpretations. The various NMR and analyses methods are all applied to unfolding of the GB1 protein for a direct comparison and to rank the various methods and their limitations. In general 2D detection and a global analysis of signals from the folded and unfolded state allow the exact determination of thermodynamic parameters of protein unfolding even if transitions are not completed. Exchangeable protons such as amides have to be analysed with care under conditions of high amide exchange, as in temperature induced unfolding, since they do not correctly monitor the disappearance of the native state. 1D detection using signals from native state methyl groups and a mixture of signals from the native and the unfolded state, leads to an underestimation of stability, if the pulse sequence includes any delay between excitation and detection, during which relaxation can occur. ^1^H–^13^C signals, especially methyl groups are the best probes to study unfolding. Urea is best suited as a denaturant, GdmCl works slightly less well, because it introduces high ionic strength and by this affects the performance of the spectrometer. Temperature induced unfolding can cause several problems and should be avoided or at least checked critically.

The here reported strengths and weaknesses of the different NMR parameters employed to study protein stability by unfolding transitions with residue resolution gives perspectives of how to design NMR experiments to follow proteins experiencing e.g. a crowded cellular environment, liquid–liquid phase separation or high pressures. As also pointed out in an recent review about NMR detected protein unfolding (Politou et al. 2021) it is not enough to interpret unfolding transitions or protein stability data of single residue but the analysis of all accessible sites will give a global picture about which core residues form the cooperative folding unit and which residues experience deviating local effects. These deviations should not per se lead to the substitution of a simple two-state folding model by a more complex one. These local effects often occur at surface exposed residues and less defined loop structures. A rigorous global analysis allows identifying residues participating to global and local conformational changes before quantifying the weak but essential intermolecular interactions, one is interested in, in order understand the molecular details of e.g. molecular crowding, LLPS or high pressure effects.

## Data Availability

All data generated or analyzed during this study are included in this published article.
